# A Dinophysistoxin 2-like Okadaic Acid Isomer Present in Bivalves and Water Column: Temporal and Spatial Distribution in the Northwest Iberian Peninsula

**DOI:** 10.3390/toxins18080345

**Published:** 2026-08-10

**Authors:** Juan Blanco, Ángeles Moroño, Fabiola Arévalo, Jorge Correa, Araceli E. Rossignoli, Juan Pablo Lamas

**Affiliations:** 1 Centro de Investigacións Mariñas (CIMA), Xunta de Galicia, Pedras de Corón s/n, 36620 Vilanova de Arousa, Spain; araceli.escudeiro.rossignoli@xunta.gal; 2 Instituto Tecnolóxico para o Control do Medio Mariño (INTECMAR), Xunta de Galicia, Peirao de Vilaxoán s/n, 36611 Vilagarcía de Arousa, Spain; amoronho@intecmar.gal (Á.M.); farevalo@intecmar.gal (F.A.); jcorrea@intecmar.gal (J.C.)

**Keywords:** diarrhetic shellfish poisoning, DSP, dinophysistoxin 2, DTX2, okadaic acid, isomer, bivalves, water, esterification, trend

## Abstract

A previously overlooked isomer of okadaic acid (OA), which is structurally related to dinophysistoxin-2 (DTX2), was identified in bivalves and water columns from the Northwest Iberian Peninsula. This isomer elutes chromatographically after DTX2 and before DTX1, with mass spectrometric fragmentation patterns distinct from those of OA. It occurs at lower concentrations than OA but at levels comparable to DTX2, showing a consistent presence since monitoring by LC-MS/MS began in 2014. The abundance of this isomer correlated more strongly with OA than with DTX2, suggesting that it originates mainly from phytoplankton species that do not produce DTX2. Seasonal patterns revealed two maxima for the isomer, aligning with OA but differing from the single autumn–winter maximum of DTX2. Spatially, the isomer followed a northeast–southwest gradient along the Galician coast, similar to the OA and DTX2 distributions. The esterification rates of the isomer in mussels are lower than those of OA but similar to those of DTX2, likely resulting in slower depuration and longer persistence. Temporal trends indicate a recent increase in the concentration and seasonality of the isomer, rendering it more conspicuous. These findings highlight the growing significance of this isomer in shellfish toxin profiles throughout the studied period, underscoring the need to determine its structure and potential toxicity to better assess risks to human health.

## 1. Introduction

**Figure 1:**
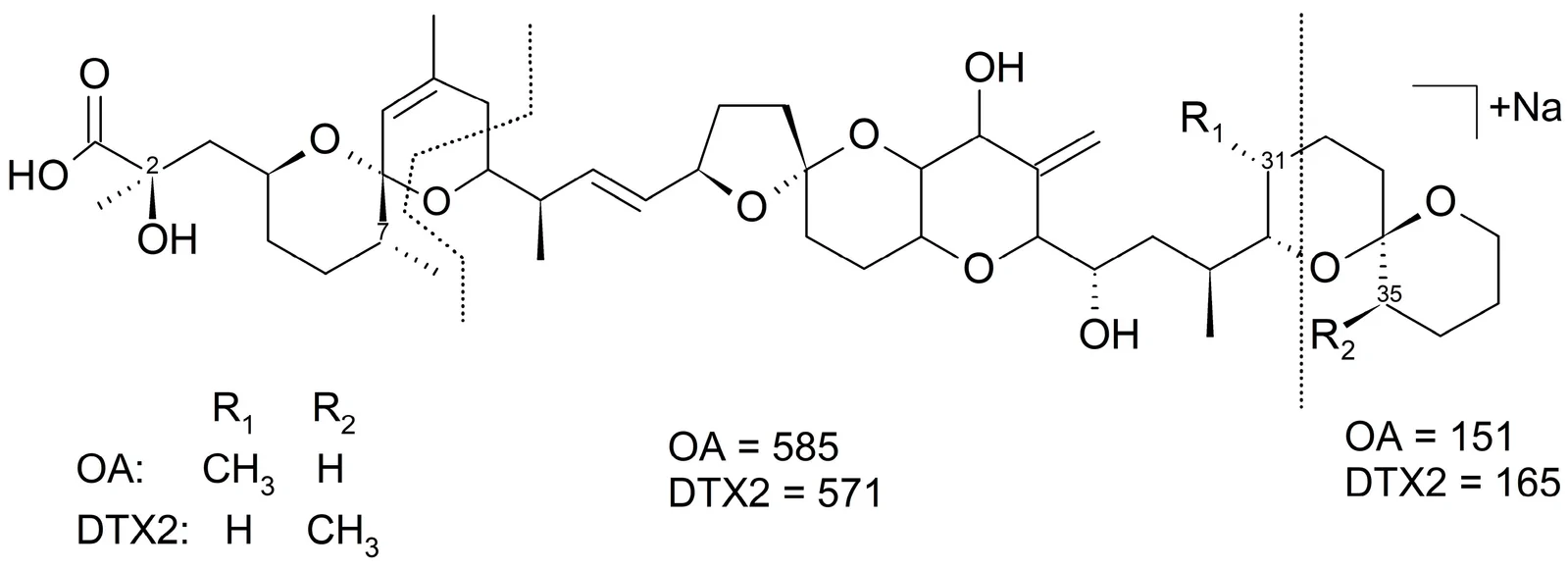
Structure of okadaic acid (OA) and dinophysistoxin 2 (DTX2) and the main CID fragments (with their corresponding *m*/*z*) of their Na adducts.

Harmful algal blooms (HABs) pose a significant threat to human health and shellfish-related economic activities worldwide. Certain HABs are caused by species that generate toxins that can accumulate in filter-feeding bivalves and other organisms, thereby impacting humans upon consumption of these organisms [1]. The main types of intoxication originating from planktonic organisms are paralytic shellfish poisoning (PSP) and diarrheic shellfish poisoning (DSP). The first can be lethal, whereas the second cannot, with symptoms mostly limited to digestive disorders, including diarrhea, nausea and vomiting [2]. To protect human health, most countries have established safe limits for the concentrations of these toxins in bivalves [3]. When these limits are exceeded, the harvesting of the organisms is prohibited, with adverse economic consequences for fishermen and aquaculture [4,5,6]. Among the known toxicities in Europe, DSP is responsible for most harvesting closures [7]. In Galicia, NW Spain (Figure 1), since monitoring exists, most mussel harvesting closures were due to this kind of toxicity [8], with years in which some culture areas were closed to the market for 295 days [9].

DSP toxicity is caused by a series of okadaic acid-related compounds [10,11,12,13]. Okadaic acid (OA) is a complex polyether compound that was originally isolated from the sponges *Halichondria okadai* and *H. melanodocia* [14]. It has a long carbon chain backbone with multiple cyclic ether rings, a carboxylic acid group at one end, and several hydroxyl groups at various positions, the most important of which is at C7. Several analogs and derivatives exist, some of which are synthesized by phytoplankton cells, such as okadaic acid, dinophysistoxin-1 (DTX1), and dinophysistoxin-2 (DTX2), which are the main toxins, and a group of compounds in which the carboxylic function of these main toxins esterifies diols or more complex compounds, which are generically named DTX4 to 7 [15,16,17,18,19]. In bivalves, and probably also in other organisms, the C7 hydroxyl is esterified with fatty acids [20,21,22], producing derivatives generically known as DTX3, even though this name was initially assigned to a mixture of esters of fatty acids with DTX1 [23].

Among the toxins in this group, OA is the most common and abundant in phytoplankton cells, followed by DTX1 and DTX2 (Figure 1). DTX1 is the primary toxin produced by some species or in some locations, and DTX2 is a secondary toxin produced by fewer species/strains than the two other toxins and at lower abundance [24]. In bivalves and other organisms, the proportions found in phytoplanktonic cells are maintained because these three toxins do not interconvert [11,25]. Several isomers of each toxin were identified. Some of these compounds have been structurally determined (e.g., 19-epi-OA and DTX-1b), while others remain unknown as an unidentified isomer that eluted after DTX2 and before 19-*epi*-DTX1, which is present in certified reference material for diarrhetic shellfish poisoning toxins [26]. Two isomers of OA that eluted after DTX2 were also found in mussels and phytoplankton from Ireland [13,27]. Their abundance relative to that of OA was low, and no subsequent studies have confirmed their presence, likely due to the use of narrow analytical windows in routine monitoring or their genuine rarity.

**Figure 2:**
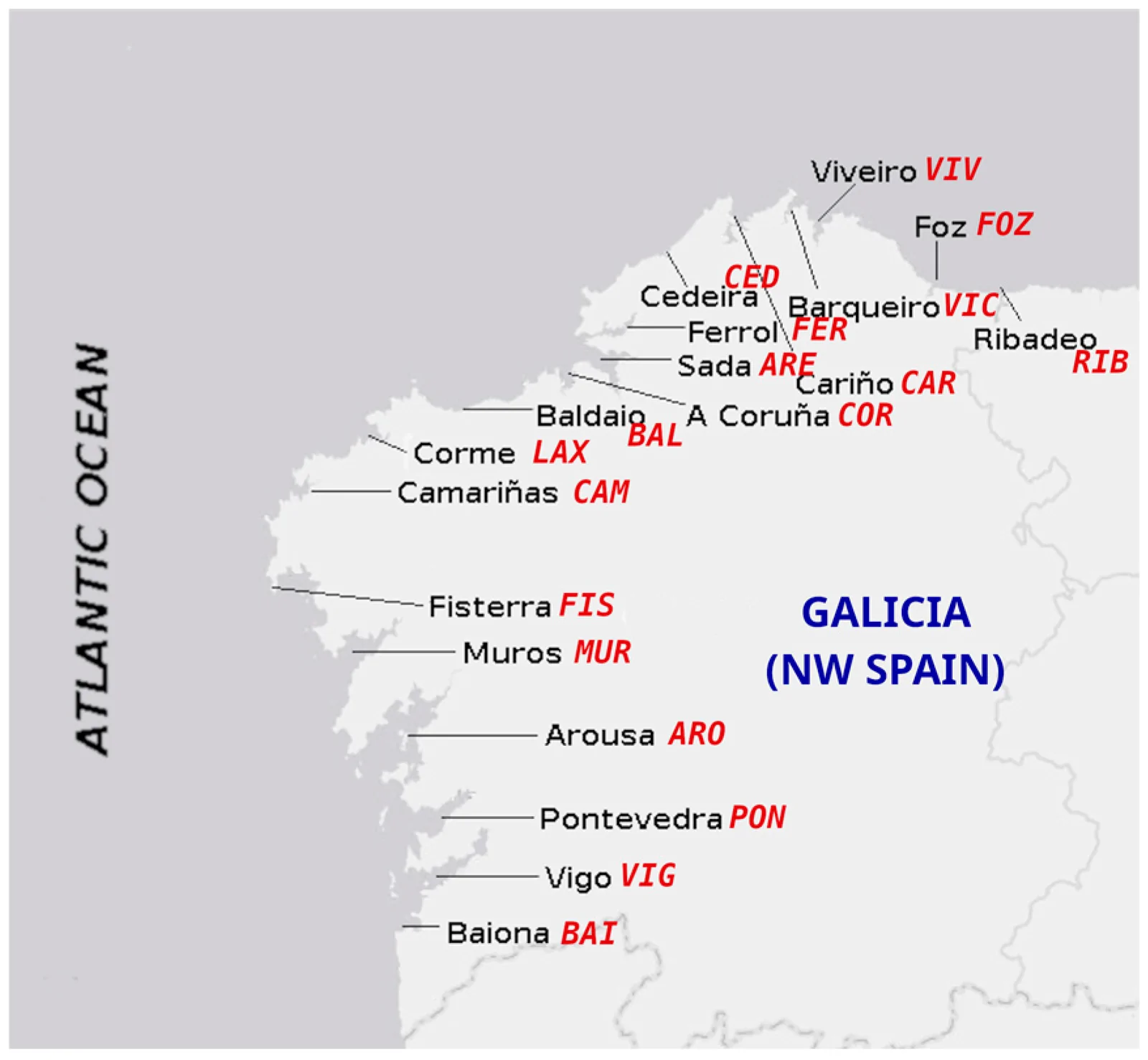
Sampling locations with their corresponding codes (red text).

In Galicia, OA is the most frequently detected toxin, followed by DTX2, with no significant presence of DTX1 [8]. OA has been mainly associated with *Dinophysis acuminata*, and both OA and DTX2 with *D. acuta* [24]. DTX2 in mussels was 10 times less frequent and 5–10 times less abundant than OA between 2014 and 2017 [8], contributing only (not being the only responsible for) to 2% of the market harvesting bans. Recently, however, during an episode mainly produced by *D. acuminata*, an OA isomer that chromatographically (reverse phase) elutes after DTX2 but before DTX1 has been detected in mussels, in proportions to OA like those frequently found for DTX2. The objectives of this study were to characterize the compound, hereafter referred to as Iso-DTX2; to determine if it was present in the water column, in addition to bivalves; and to determine its temporal trend, spatial distribution, esterification behavior, and its contribution to DSP toxicity in the NW Iberian Peninsula (Figure 2).

## 2. Results and Discussion

### 2.1. Chromatographic and Mass Spectrometric Characteristics

**Figure 3:**
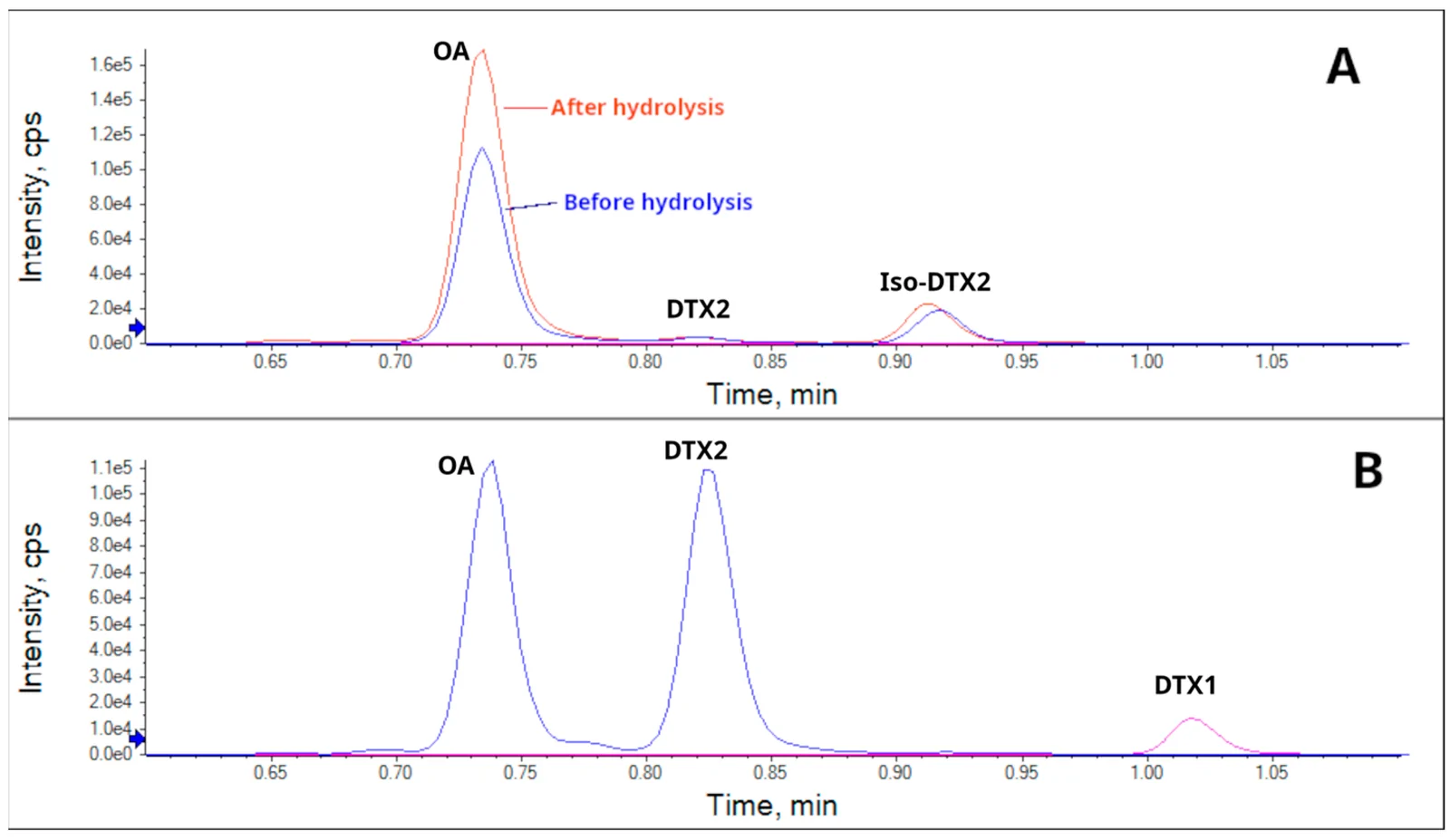
Extracted ion chromatograms using the transitions 817.5 > 255.1 for DTX1 and 803.5 > 255.1 for OA, DTX2 and Iso-DTX2 of a mussel sample (**A**) and a reference material containing OA, DTX2, and DTX1 (**B**). For the mussel sample, both the free forms (before hydrolysis) and the free plus conjugated forms (after hydrolysis) are shown.

By inspecting the chromatograms of mussel extracts and water samples from Ría de Pontevedra (Galicia), obtained using two characteristic transitions of OA and DTX2, 803.5 > 255.1 and 803.5 > 563.3, a chromatographic peak was found that eluted after DTX2 but before DTX1 (Figure 3).

**Figure 4:**
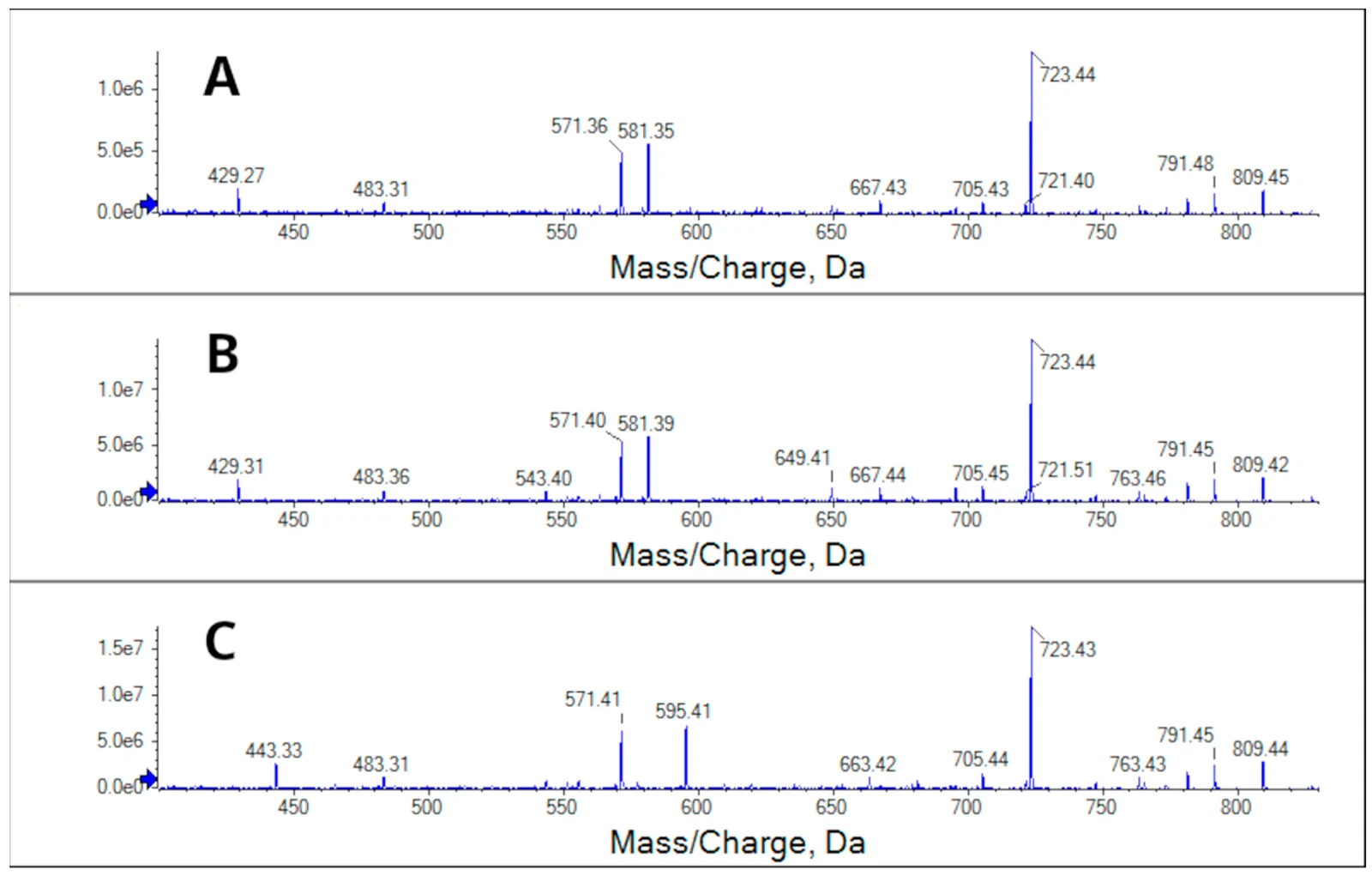
CID fragmentation spectra in the range of 100–400 *m*/*z* of the Na adducts of Iso-DTX2 (**A**), DTX2 (**B**), and OA (**C**) using positive ionization (collision energy: 82). Iso-DTX2 from a mussel sample, and OA and DTX2 from certified reference materials.

Its fragmentation cannot be distinguished from that of OA in either the positive or negative ionization mode (Figures S1–S3); however, it differs when using the Na adduct (Figure 4). In that case, the fragments 581 and 429 *m*/*z* were observed, which are characteristic of DTX2, instead of 596 and 443 *m*/*z*, which are characteristic of OA.

**Figure 5:**
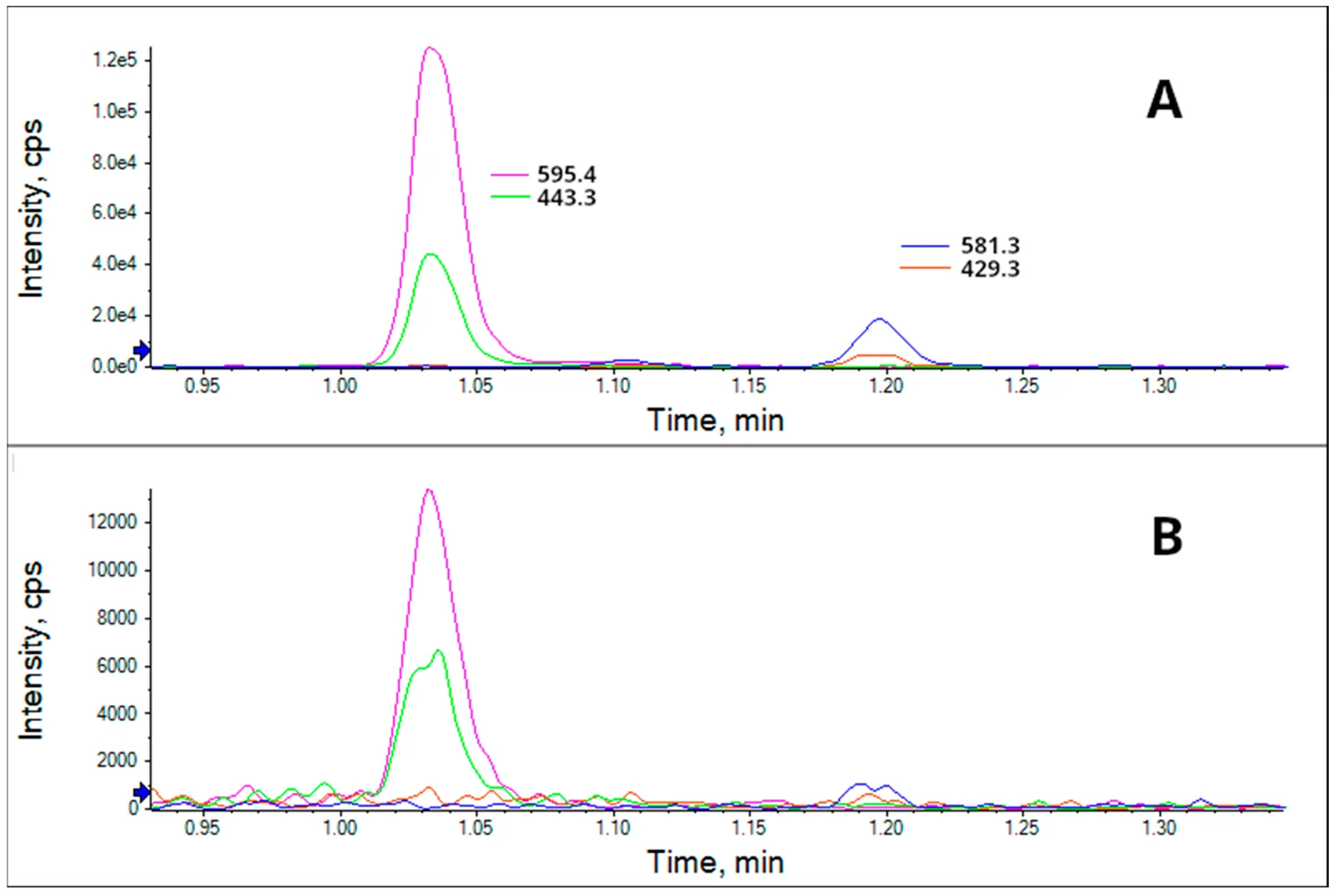
Chromatograms of a mussel sample from the Ría de Pontevedra (**A**) and particulate matter in the water from the Ría de Pontevedra (**B**) using the differential fragments of the Na adducts of DTX2 and OA in positive ionization mode.

By multiple reaction monitoring of the differential fragments, Iso-DTX2 was detected in both mussel and water samples (Figure 5).

The presence of these fragments that differ from okadaic acid, together with two additional differential fragments, 151 *m*/*z* in OA and 165 *m*/*z* in DTX2 and iso-DTX2 (obtained by MS^3^ from the 723 *m*/*z* fragment of the Na adduct (827.5 *m*/*z*)) (Figure S4), indicates that the methyl group is not placed in C31 as in OA and could be located in C35 as in DTX2. Carey et al. [28] proposed a CID fragmentation pattern for the Na adducts of OA and DTX2 in which the observed difference in those three pairs of ions is due to the presence or absence of a methyl group in C35. In DTX2 and Iso-DTX2, the methyl group should be part of the 165 *m*/*z* fragment; however, unfortunately, the fragmentation of that ion, which would have allowed us to determine whether the methyl group was placed on the same carbon atom as in DTX2, was not possible. Iso-DTX2 has the same retention time as an unidentified isomer present in CRM-DSP-Mus-c when both are injected on the same day using the same equipment (Figure S5).

Two OA isomers that elute between OA and DTX1 have been described in Ireland: DTX2B and DTX2C [13,27]. Unfortunately, the lack of a fragmentation spectrum for the first compound and the lack of a comparison of its fragmentation spectrum with that of OA in the case of the second, together with the differences in fragmentation by different mass spectrometers, make it impossible to definitively identify whether this compound is DTX2B, DTX2C, or a distinct isomer; therefore, we have named it Iso-DTX2. Regardless of structural identity, this study provides the first comprehensive characterization of its temporal and geographical distribution as well as its ecological behavior in the NW Iberian Peninsula, where it represents a previously unrecognized component of DSP toxicity.

### 2.2. Contribution of Iso-DTX2 to Total OA-Group Toxin Content

**Figure 6:**
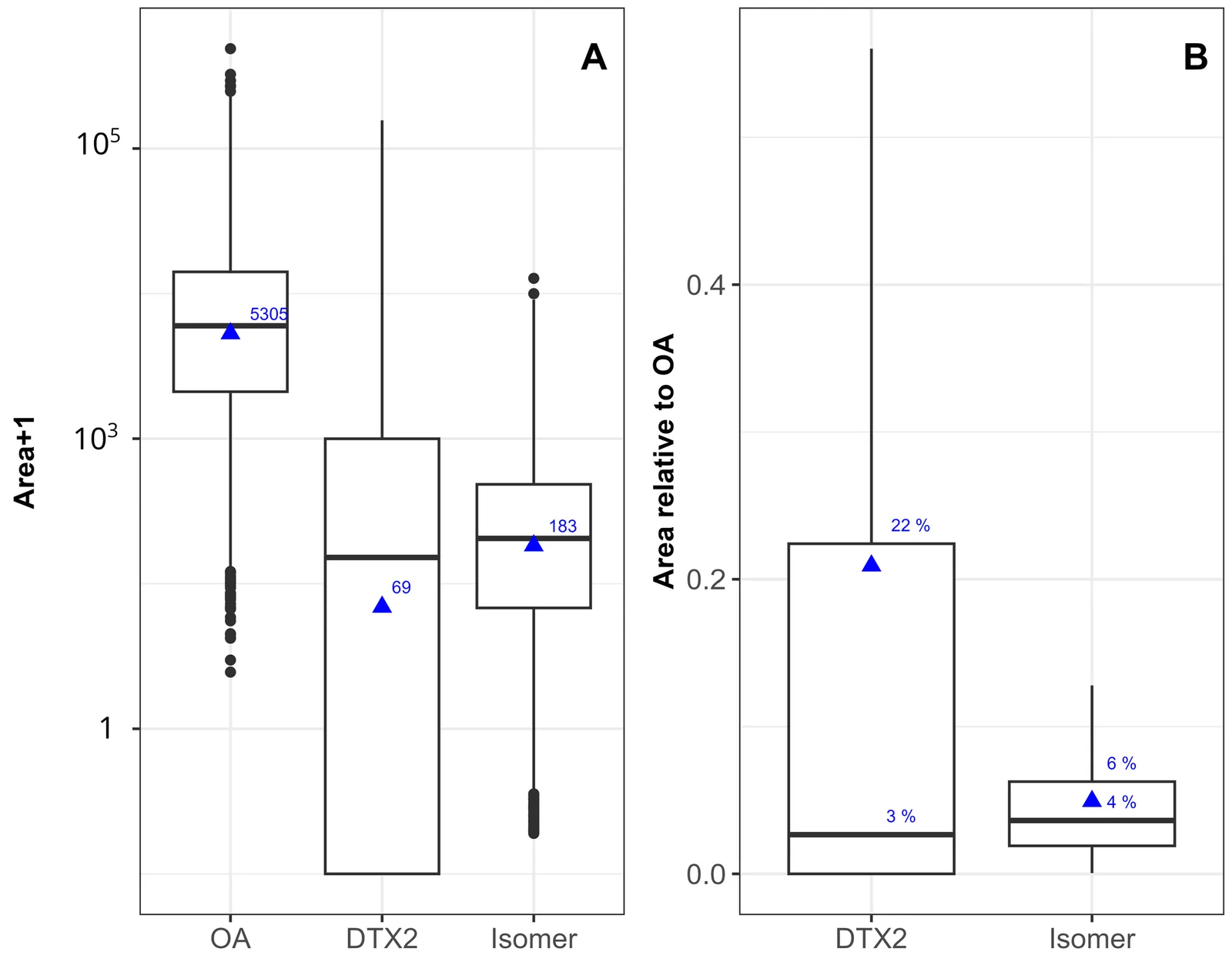
Concentration (measured as chromatographic peak area) of Iso-DTX2 and DTX2 relative to OA in absolute units of peak area (**A**) and as the ratio of peak areas to OA (**B**). The box boundaries correspond to the first (Q1) and third (Q3) quartiles, the central line indicates the median, the whiskers extend to the most extreme values within 1.5 × IQR from Q1 and Q3, and the individual points beyond the whiskers represent the outliers. Triangles indicate the means.

In bivalves, Iso-DTX2 is found in both hydrolyzed and non-hydrolyzed samples at concentrations that are consistently lower than OA. However, its concentration is not always less than that of DTX2. The average area of its peaks was 183, which was clearly lower than 5305 corresponding to OA (approximately 3.5%), but it was closer to 69, the area of DTX2 (Figure 6A).

The median contributions of Iso-DTX2 and DTX2 in bivalves (Figure 6B) were 4% and 3% of that of OA, respectively. However, the dispersion of the percentages of the isomer was substantially smaller than that of DTX2, with the 75% percentile being 6% for the isomer and 22% for DTX2.

No data on the relative importance of DTX2B and DTX2C are available for Ireland. However, in the articles that described them, they were mentioned as minor components [13,27]. In contrast, our data reveal that Iso-DTX2 accounts for an average of 4% of OA levels in the NW Iberian Peninsula. This finding implies that Iso-DTX2 may hold toxicological and regulatory relevance in this region, particularly in light of the observed increasing trend over time. Future efforts should focus on acquiring reference materials for this toxin and assessing its toxicity in comparison to OA to safeguard public health.

### 2.3. Relationship with OA and DTX2

**Figure 7:**
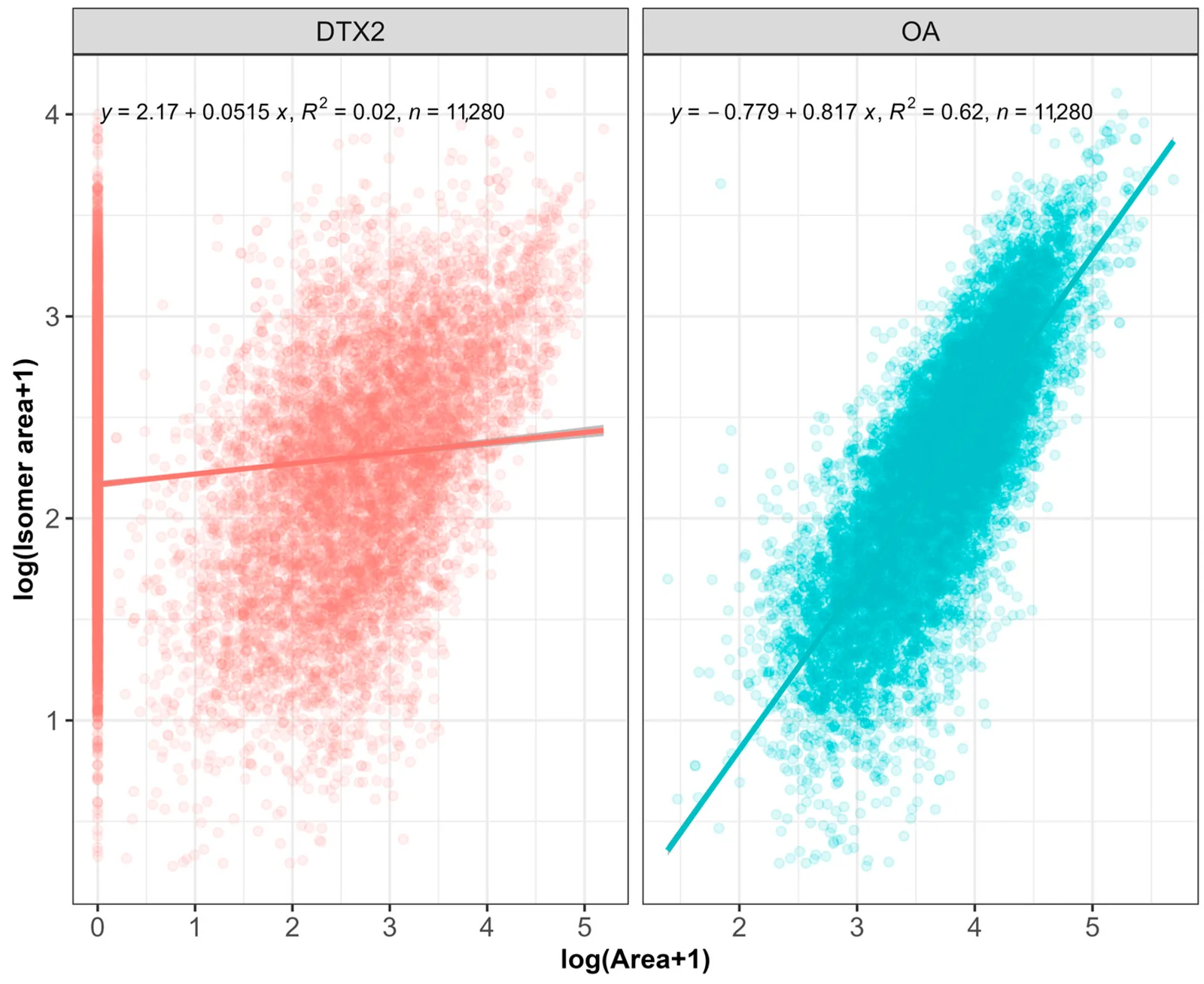
Relationship of Iso-DTX2 with DTX2 and OA, with the corresponding regression equations, coefficient of determination (R^2^), and number of observations (n).

Iso-DTX2 concentrations appeared to be more closely related to OA than to DTX2. A large number of Iso-DTX2 detections were observed in samples that did not contain DTX2. The R^2^ of the regression with DTX2 (log-log transformed) was much smaller (0.02) than that with OA (0.62) (Figure 7).

The correlation between OA and DTX2 makes it impossible to unequivocally determine whether Iso-DTX2 is produced by DTX2-producing phytoplankton species (Table S1). As the occurrence of mixed blooms containing both DTX2-producing and non-DTX2-producing phytoplankton species is frequent in the area, only direct analysis of isolated phytoplankton cells or cultures can, in the future, definitively establish species-specific production. However, the much lower regression coefficient and the high number of cases in which Iso-DTX2 was present in samples in which DTX2 was not detected suggest that DTX2-producing phytoplankton species do not produce the isomer or produce it in substantially lower amounts than those that do not produce DTX2.

### 2.4. Esterification

**Figure 8:**
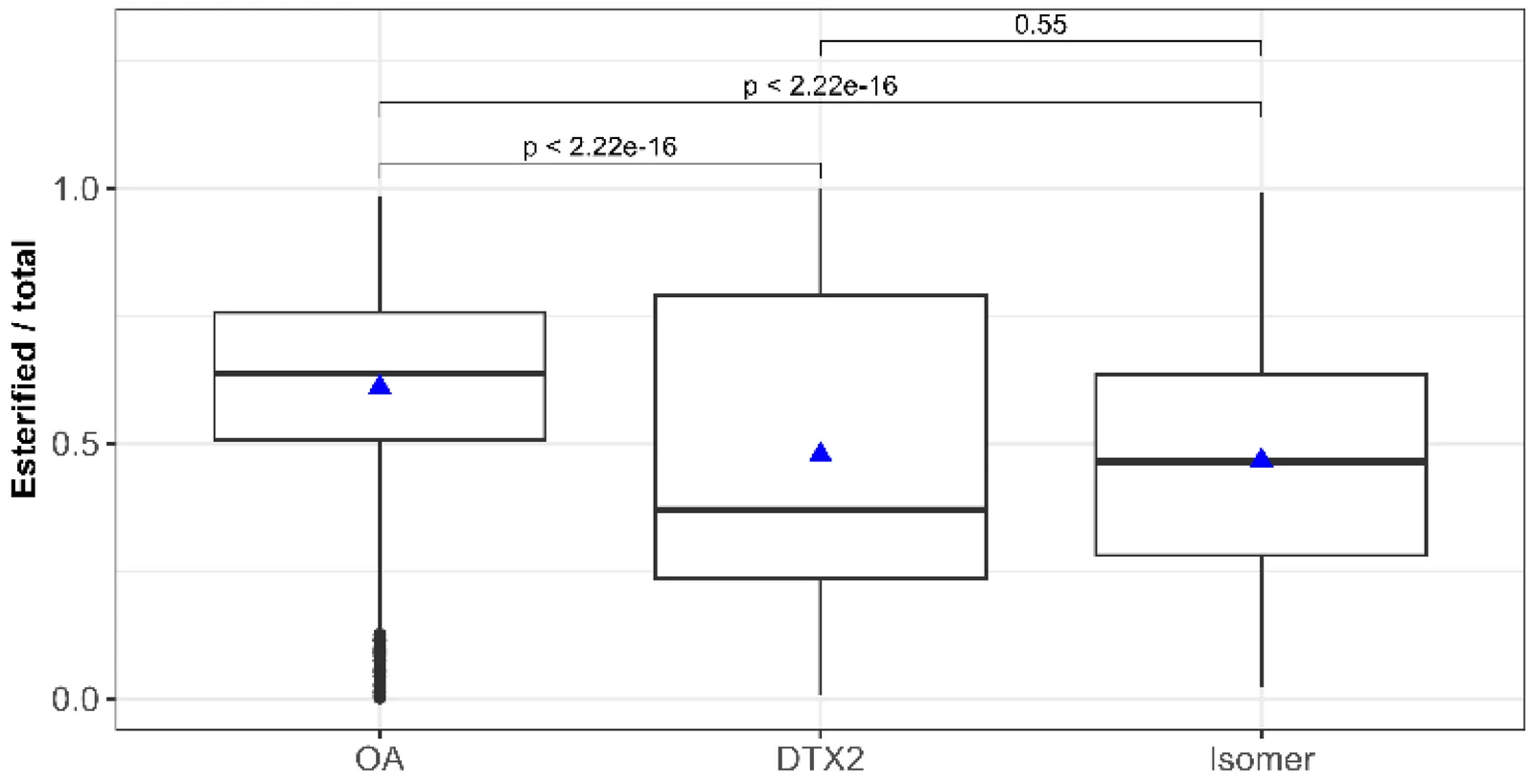
Proportion of esterified forms of the three toxins found in mussels and statistical significance (Wilcoxon test) of the differences between them. The box boundaries correspond to the first (Q1) and third (Q3) quartiles, the central line indicates the median, the whiskers extend to the most extreme values within 1.5 × IQR from Q1 and Q3, and the individual points beyond the whiskers represent the outliers. Triangles indicate the means.

Iso-DTX2 is esterified by mussels in a manner similar to DTX2 [8,29,30,31]. That is, in a smaller proportion than OA, in such a way that during the depuration period, the proportion between Iso-DTX2 and OA increases depending on the accumulation phase (Figure S6). The proportion of the toxin in the esterified form relative to the total was significantly different between Iso-DTX2 and OA but not between Iso-DTX2 and DTX2 (Figure 8).

### 2.5. Temporal Variability and Trend

**Figure 9:**
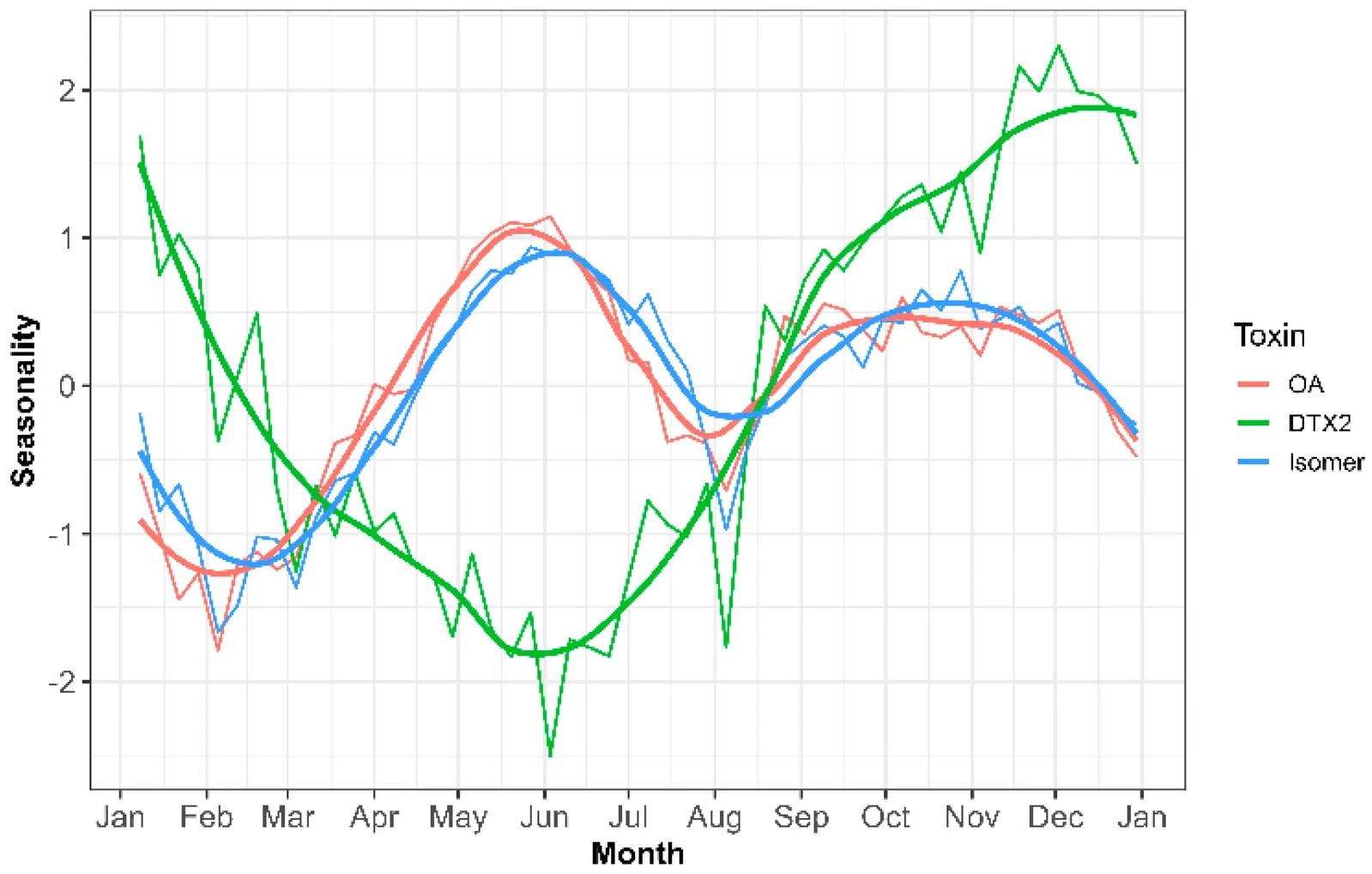
Seasonal component of the concentrations of OA, DTX2 and Iso-DTX2 in bivalves (narrow lines), obtained by time series decomposition with the R stl function. Thick lines represent the loess-smoothed correspondents.

Iso-DTX2 has been detected since LC-MS/MS was first used to monitor lipophilic toxins in the Galician Monitoring Programme, in 2014. It presents a clear seasonal pattern that coincides well with that of OA but not with DTX2 (Figure 9). It presents two maxima, one in spring–summer and another in autumn, differing from DTX2, which presents only one maximum in autumn–winter.

The observed pattern also points to a strong association with non-DTX2-producing phytoplankton species because of a different pattern of DTX2, even in the second maximum. The main *Dinophysis* species that produces DTX2, *D. acuta*, typically blooms in the area once a year in autumn [32], whereas the other main *Dinophysis* species in the area, *D. acuminata*, has a wider growing season, allowing the two species to co-occur [32,33]. Unequivocal assignment of Iso-DTX2 to a precise species will require the study of isolated cells or cultures, which is beyond the scope of this study.

**Figure 10:**
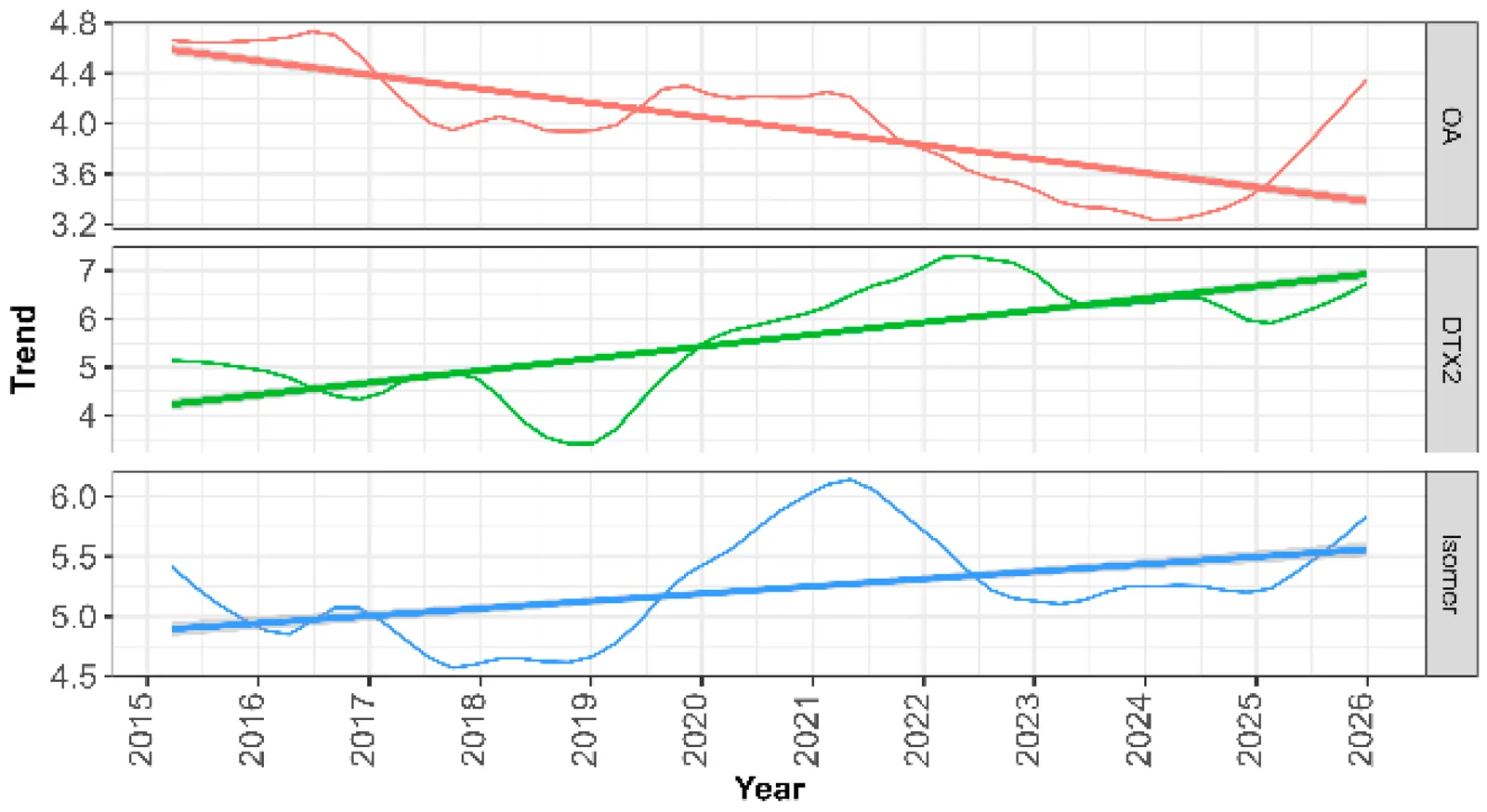
Deseasonalized trend of the concentration of OA, DTX2, and Iso-DTX2 in bivalves. The straight lines are linear regression lines fitted to show the general trend over the study period.

Iso-DTX2 showed a general ascending deseasonalized trend, characterized by an initial period from 2015 to 2019, which was nearly constant, followed by a maximum in 2021, another nearly constant period between 2024 and 2025, and a final rise in 2026 (Figure 10). The overall trend was consistent with that observed for DTX2 but differed from that of OA.

**Figure 11:**
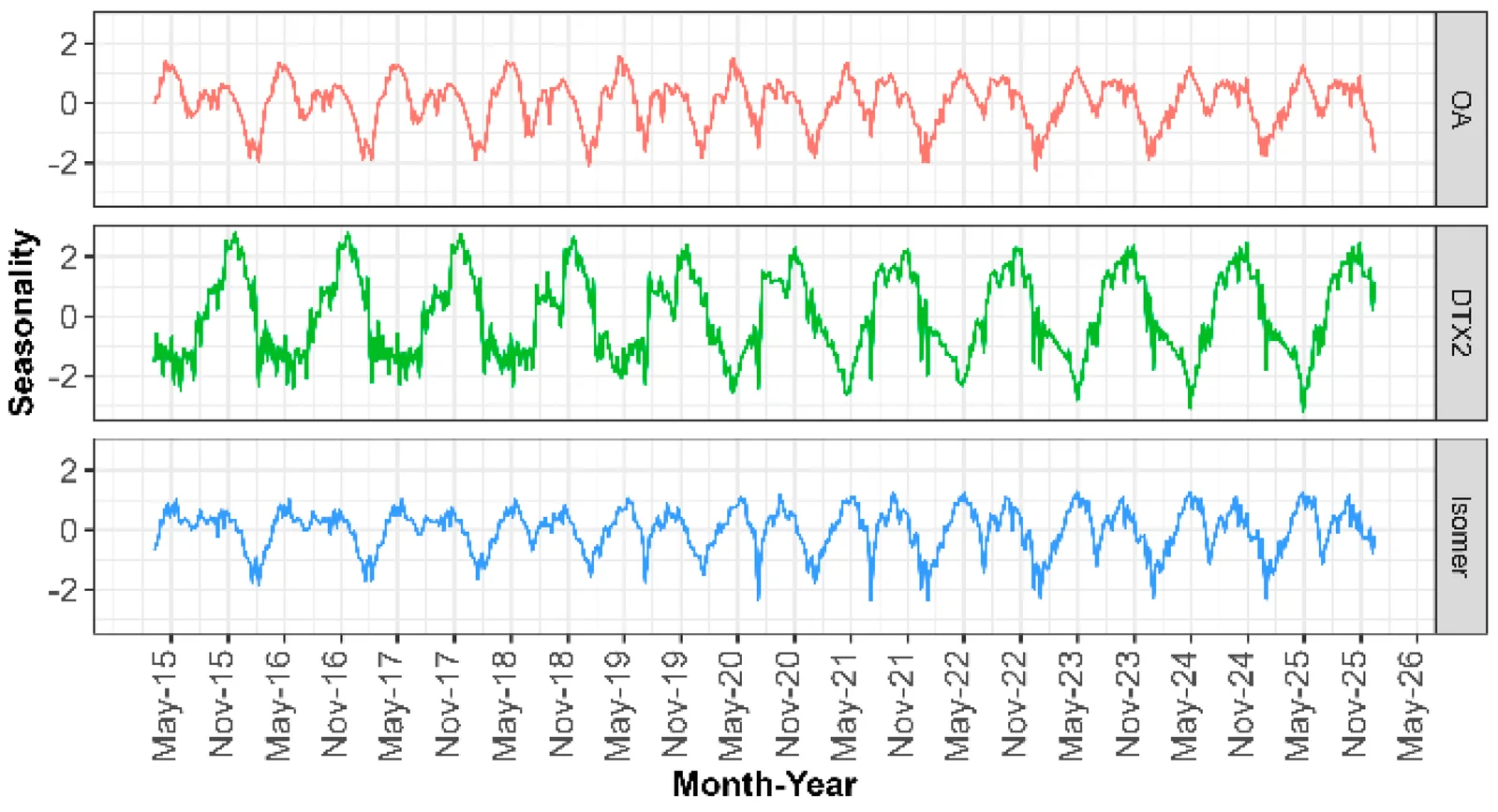
Seasonal patterns of OA, DTX2, and Iso-DTX2 obtained by allowing for different seasonality between years.

Seasonality, particularly for Iso-DTX2, varied throughout the study period. A clear increase in seasonality can be observed, but without an appreciable change in the months in which maxima occurred. DTX2 seasonality also increased, but only slightly, with a clear shift towards autumn–summer in the time at which maxima occurred. It seems that the spring and summer–autumn amounts of the isomer started to be separated by a valley coinciding with the expansion of DTX2 towards summer (Figure 11). Some changes in chlorophyll a and primary productivity have been found in the area in recent years [34], and the underlying causes are worth studying.

### 2.6. Spatial Variability

**Figure 12:**
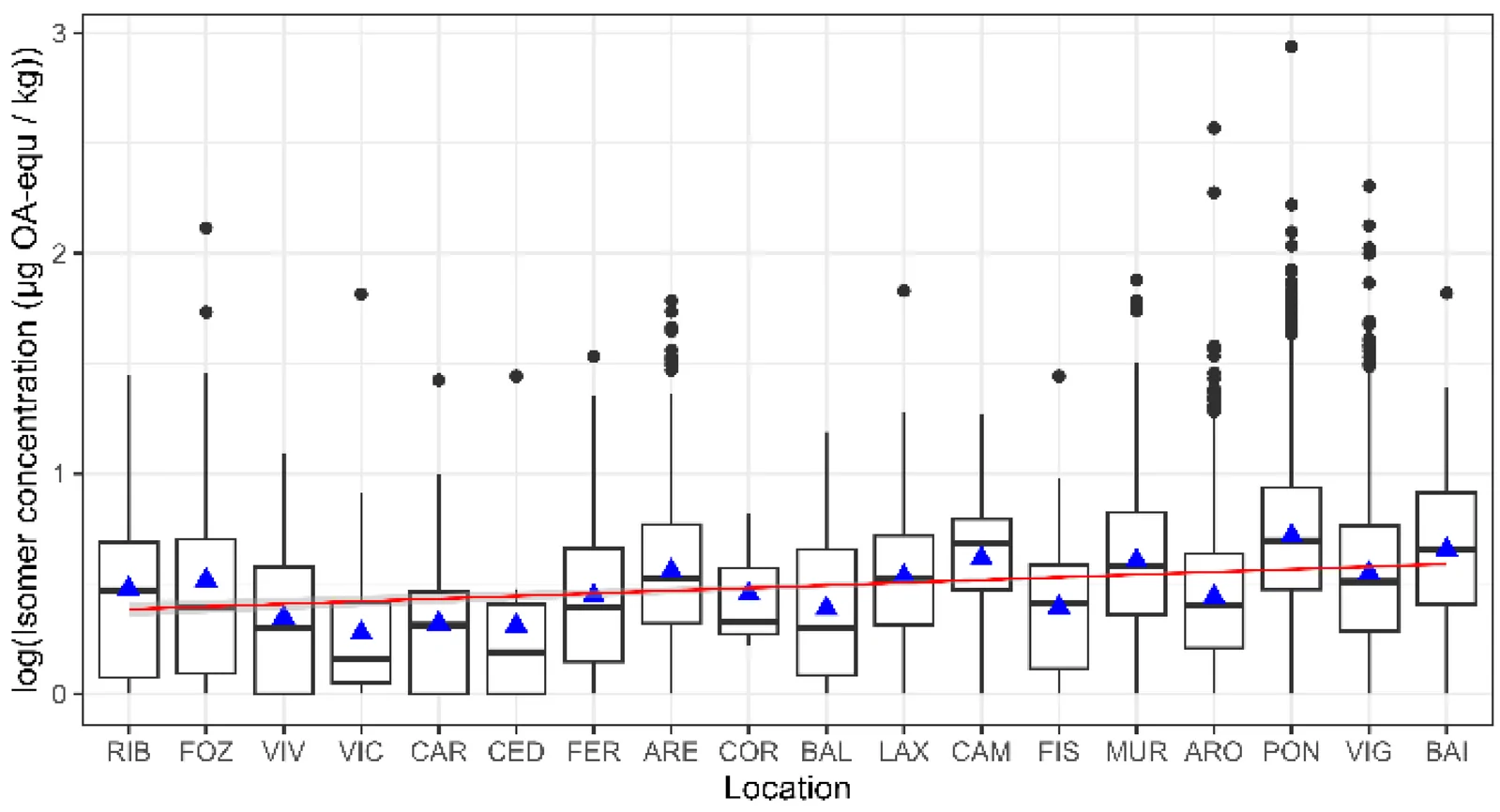
Estimated concentrations of Iso-DTX2 in mussels along the Galician coast and regression fit using the northeast–southwest order of the sampling locations as an independent variable. The box boundaries correspond to the first (Q1) and third (Q3) quartiles, the central line indicates the median, the whiskers extend to the most extreme values within 1.5 × IQR from Q1 and Q3, and the individual points beyond the whiskers represent the outliers. Triangles indicate the means.

Iso-DTX2 levels varied along the Galician coast (Figure 12). To avoid possible biases due to the main molluscan species sampled from each area, only mussels (raft-cultured and wild populations) were used for the analysis. A trend from northeast to southwest was observed, which coincided with the same trend for OA and DTX2.

**Figure 13:**
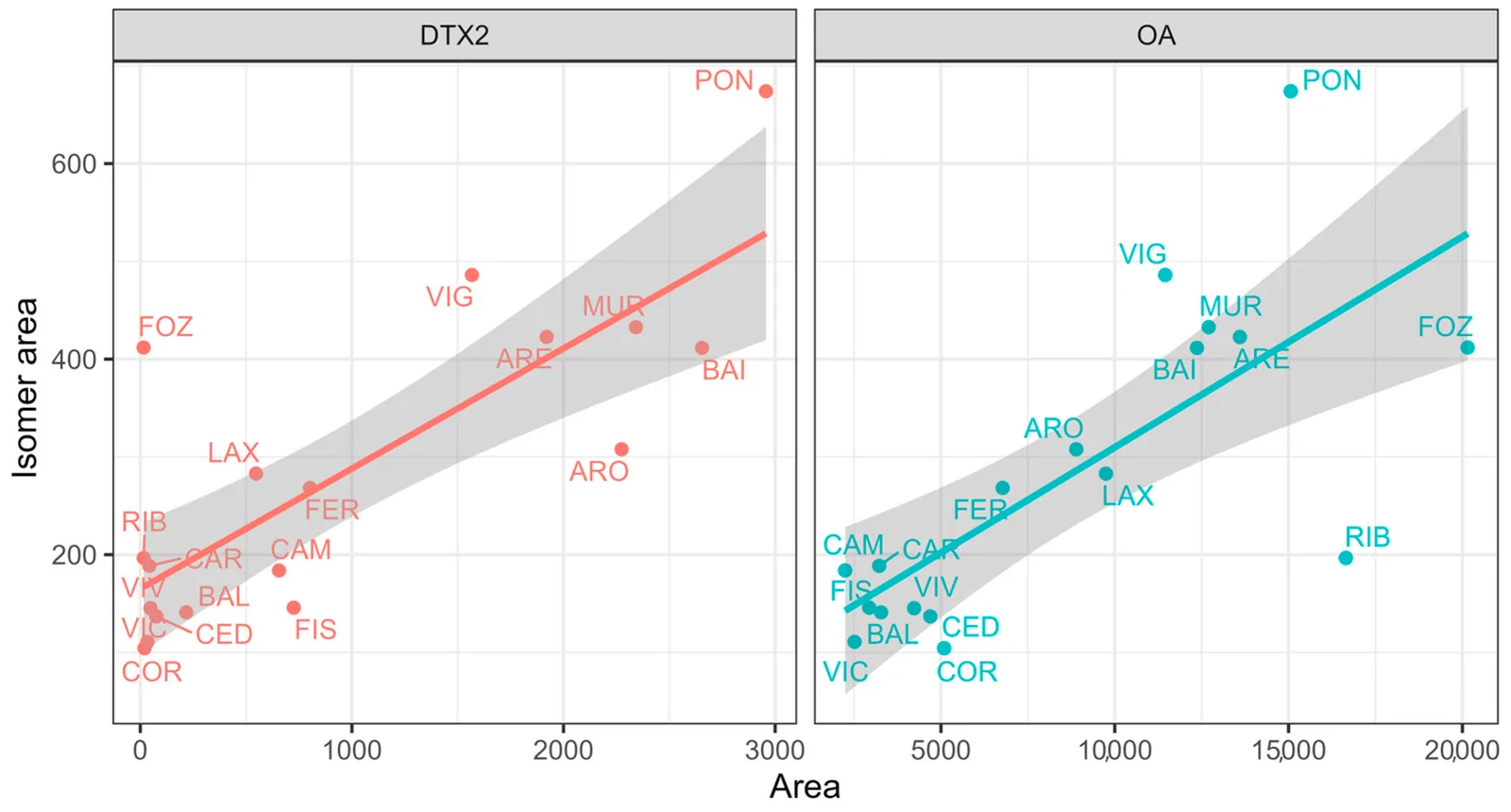
Relationship between the average areas for each sampling location of Isomer, OA and DTX2. The labels indicate the location.

The relationship between Iso-DTX2 and both OA and DTX2, when averaged by location, was generally good, suggesting that the observed trend was not mainly due to any special characteristic of the local populations. However, a few locations, especially Pontevedra (PON) and Vigo (VIG), showed considerable differences from the general trend (Figure 13). In these areas, the harvesting prohibitions by OA and DTX2 are usually longer than in other locations [9].

### 2.7. Bivalve Mollusks Interspecific Variability

**Figure 14:**
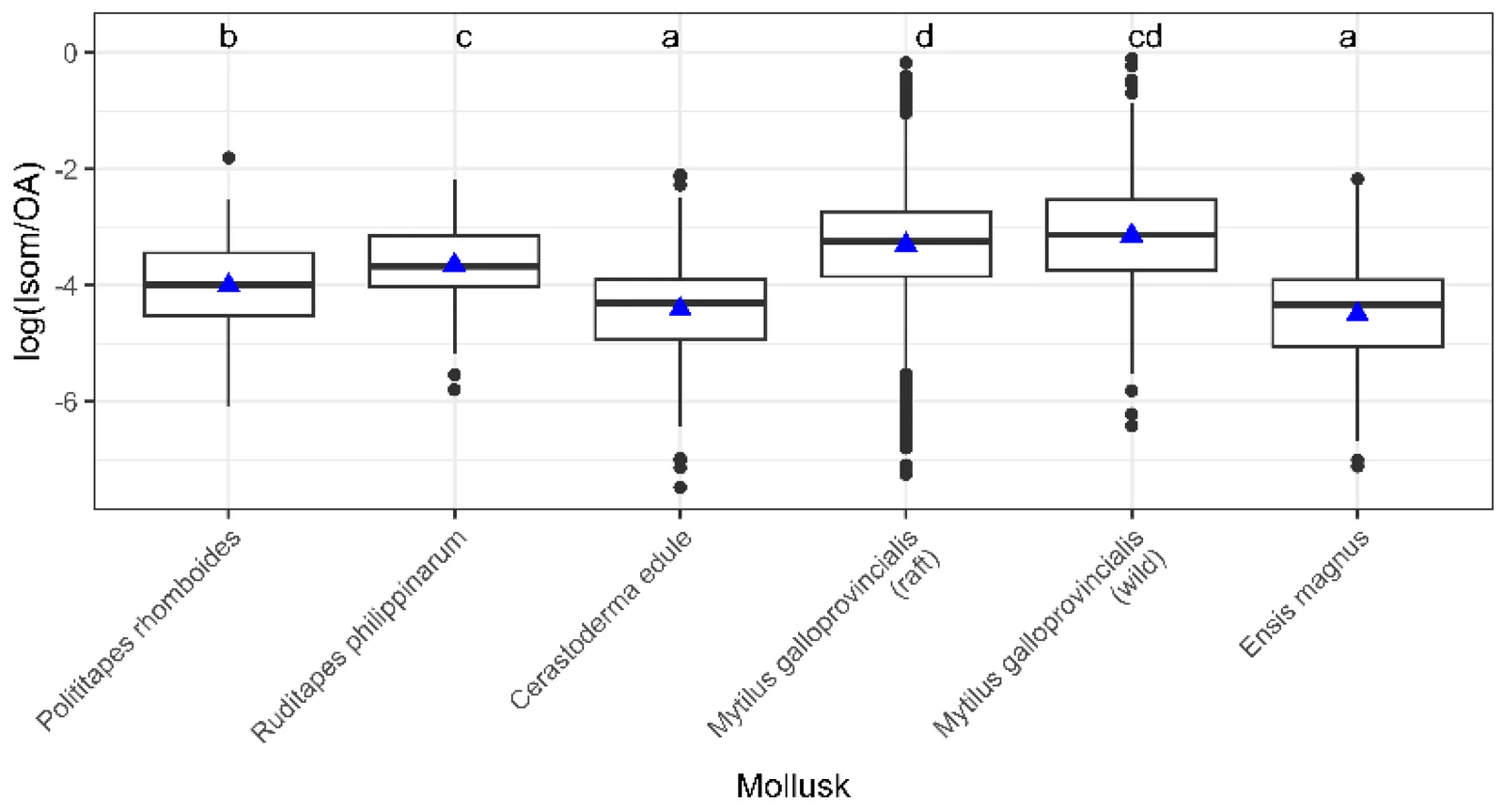
Ratio of the peak areas of Iso-DTX2 and OA in the mollusk with 100 or more determinations. Letters in the upper line are the groups defined by Tukey’s a posteriori test. The box boundaries correspond to the first (Q1) and third (Q3) quartiles, the central line indicates the median, the whiskers extend to the most extreme values within 1.5 × IQR from Q1 and Q3, and the individual points beyond the whiskers represent outliers. Triangles indicate the means.

The highest proportion of Iso-DTX2 relative to okadaic acid was recorded in mussels, especially in wild populations (Figure 14), probably due to a lower depuration rate, which in turn is due to a lower esterification rate. The okadaic acid group toxins need to be esterified with fatty acids to be excreted from bivalve digestive gland cells [29]; therefore, toxins that are esterified more slowly are depurated at a lower rate, as happens in *M. galloprovincialis* with DTX2 [8,35]. Other bivalve species esterify and likely depurate DTX2 and OA at approximately the same rate. Consequently, an accumulation of DTX2, and probably Iso-DTX2, relative to OA does not occur.

## 3. Conclusions

An isomer of okadaic acid, which is of increasing importance in the NW Iberian Peninsula, was detected. It was found in bivalves and water samples, suggesting that it is not a product of biotransformation in bivalve mollusks. Based on CID fragmentation, it appears to be structurally more related to DTX2 than to OA. Chromatographically (reverse phase and alkaline mobile phases), it elutes after DTX2 but close to it, and consequently, it can be lost if the detection window is very narrow, or it can be misidentified as DTX2. It is more associated with OA than with DTX2, both by its seasonal pattern and because it frequently appears when DTX2 is not present, suggesting that DTX2-producing phytoplankton species are not its main source. The isomer has approximately the same importance as DTX2, but it seems to be increasing in both its overall trend and seasonality. Its esterification rate seems to be lower than that of OA and close to that of DTX2, which is likely to lead to lower depuration rates, increasing its importance for human safety. Mussels are the species that have the highest proportion of this compound, probably because they have lower esterification rates than other mollusks. This study provides the first comprehensive characterization of the temporal and geographical distribution of the DTX2 isomer Iso-DTX2 as well as its ecological behavior in the NW Iberian Peninsula, where it represents a previously unrecognized component of DSP toxicity. Iso-DTX2 should be considered for the management of bivalves, both because it can be a toxicity source not previously considered, and because it can be taken for DTX2 in the analyses if precautions are not taken to quantify them independently.

## 4. Materials and Methods

### 4.1. Sampling

Between January 2014 and December 2025, samples for LC-MS/MS analysis were gathered according to the action plan of Intecmar, which required at least weekly sampling from each production area during the permissible harvesting periods. Mussel samples were collected from three different depths on raft culture ropes across 26 production areas, with samples from these depths typically combined into a single sample for analysis. In 16 areas where wild populations of various bivalve species existed but no raft mussel culture was present, wild mussels were collected weekly. Other bivalve species were sampled only when EU-regulated toxins were detected in the mussels. Owing to this sampling approach and the local prevalence of the species studied, mussels were the most frequently sampled species, followed by the cockle, *Cerastoderma edule*. Other species, such as the razor clam *Ensis magnus* and the clams *Polititapes rhomboides* and *Ruditapes philippinarum*, were analyzed less frequently. All species were tested for total OA, DTX1, and DTX2; however, only a subset was examined for free forms of the toxins.

Plankton samples were obtained from the Ría de Pontevedra in 2026 using a 20 µm mesh plankton net, which effectively retains the OA-producing *Dinophysis* species that bloom in the area (typically *D. acuminata* and *D. acuta*). The concentrates obtained were retained in Whatman GF/F glass fiber filters, which were extracted with 4 mL of MeOH and subjected to ultrasound for 15 min. The extracts were clarified by centrifugation at 4000× *g* for 10 min, and an aliquot was hydrolyzed following the same protocol as for the bivalve samples.

### 4.2. Analysis

#### 4.2.1. Extraction and Hydrolysis

Toxin extraction from bivalves was conducted according to the standardized operating procedures of the EU Reference Laboratory [36] with minor changes. This process involved opening the bivalves and discarding their shells. A homogenized sample of 100–150 g of meat was prepared, from which a 2 g subsample was extracted. This subsample was extracted with 9 mL of methanol (MeOH, several suppliers throughout the studied period) by vortexing the suspension for one minute. The mixture was then centrifuged at 2000× *g* for ten minutes, and the supernatant was collected. The remaining pellet was resuspended by vortexing the suspension for one minute in 9 mL MeOH and centrifuged under the same conditions. The resulting supernatant was combined with the initial supernatant, and the total extract volume was adjusted to 20 mL using MeOH. The total concentration of toxins in the OA group was determined by alkaline hydrolysis prior to analysis. Specifically, 625 μL of 2.5 M NaOH was added to a 5 mL aliquot of the methanolic extract, followed by vortexing for 30 s, weighing, and heating at 76 ± 4 °C for 40 min. The hydrolysate was then allowed to cool to room temperature, weighed to assess solvent loss due to evaporation, neutralized with 62 (several suppliers throughout the studied period) 5 μL of 2.5 M HCl (several suppliers throughout the studied period), and vortexed again. An aliquot of the neutralized hydrolysate was filtered through a 0.22 μm syringe filter and diluted in a 5/8 ratio with MeOH [36]. In a subset of samples, the extract was analyzed without hydrolysis to quantify the OA group toxins in free form.

#### 4.2.2. LC-MS/MS

The methodology employed in this study was adapted from the approaches outlined by Gerssen et al. [37] and Regueiro et al. [38] and subsequently optimized and validated at Intecmar in accordance with the Standard Operating Procedure (SOP) of the EU Reference Laboratory for the determination of marine lipophilic toxins in mollusks via LC-MS/MS, version 4 [36]. This method was accredited under the UNE-EN ISO/IEC 17025 standard (Accreditation No. 160/LE 394). Chromatographic separation was conducted using an Acquity UPLC BEH C18 column (2.1 × 100 mm, 1.7 μm) (Waters, Barcelona, Spain) at 45 °C, employing a binary gradient of the mobile phase. Mobile phases A and B consisted of 6.7 mM NH_4_OH (pH 11) and 90% MeCN with 6.7 mM NH_4_OH, respectively. The gradient started with 25% B and was maintained for 0.66 min, followed by a linear increase to 95% B at 3.3 min, which was sustained until 5.28 min. Subsequently, the composition was linearly reverted to the initial state over two minutes and maintained until 9 min to equilibrate the column for subsequent injections. Three LC systems were used: one Waters Acquity UPLC system (coupled to a Waters Xevo TQ MS) and two Waters Acquity H-class systems (coupled to Waters Xevo TQ-S). Toxin detection was achieved using three triple quadrupole mass spectrometers: one Xevo TQ MS (Waters, Barcelona, Spain) and two Xevo TQ-S (Waters, Barcelona, Spain), all equipped with ESI interfaces. The systems operated in both the positive and negative ionization modes. For the TQ MS, a capillary voltage of 1 V was applied, whereas for the two TQ-S, a voltage of 2 V was used. All other source operating parameters were consistent across both MS models: solvation temperature of 450 °C, solvation gas flow of 850 L·h^−1^, and cone gas flow of 150 L·h^−1^. Although the method encompassed other lipophilic toxins, this study focused solely on the transitions 803.5 > 255.2 for OA, DTX2, and the isomer. The transition 817.5 > 255.2 corresponding to DTX1 was also checked but only to verify that the retention time of the isomer was between those corresponding to DTX2 and DTX1.

Collision-Induced Dissociation (CID) fragmentation studies were performed using a SCIEX EXION chromatographic system (SCIEX, Framingham, MA, USA) with a Phenomenex Gemini-NX C18 50 × 2.1 mm, 2.7 µm column, coupled to a SCIEX QTRAP 6500+ operated in positive and negative ionization, and using the Enhanced Product Ion (EPI) mode.

### 4.3. Chromatogram Processing

Chromatogram files obtained using MassLynx software v.4.2 (Waters) were converted to mzML format using Proteowizard msconvert v.3 [39]. These files were imported into R using the MSnbase 2.39.1 package [40], the transition 803.5 > 255.5 selected, and the chromatogram smoothed using MsCoreUtils v.3.23 [41]. Chromatographic peaks were then detected, refined, and integrated using the findChromPeaks function of the XCMS package v.4.10.1 [42,43] with the centWave detection method.

Data obtained from the quality controls of each sample batch, which contained OA and DTX2, were used to determine the retention times of the two toxins and estimate Iso-DTX2 in each batch. Estimates of the areas corresponding to each peak were filtered to discard those for which the estimates of the two replicate injections differed by more than 20% and for extreme values. The areas of the two injections were averaged and analyzed statistically.

Peak areas were used instead of concentrations in most comparisons because they are a more reliable and direct way of comparing the amounts of the isomer and those of OA and DTX2 in every individual chromatogram. The limits of detection were defined in centWave and were the same for all mass spectrometers.

### 4.4. Statistical and Representation Methods

All plotting and statistical analyses were performed using the R statistical software (version 4.5.1) [44]. The ggplot2 v.4.0.3 [45], GGally v.2.4.0 [46], scales v.1.4.0 [47], patchwork v.1.3.2 [48], ggpmisc v.1.0.0 [49], and ggpubr v.0.6.2 [50] packages were used for plotting. ANOVAs, linear regression, Wilcoxon, Tukey HSD tests, and time series analysis (with the stl function (Seasonal Decomposition of Time Series by Loess)) were performed using R v.4.6.1 base.

## Data Availability

The raw data supporting the conclusions of this article will be made available by the authors upon request.

## Supplementary Materials

The following supporting information can be downloaded at: https://www.mdpi.com/article/10.3390/toxins18080345/s1, Figure S1: CID fragmentation spectra in the range 300–805 *m*/*z* of Iso-DTX2 (A) (from a mussel sample), DTX2 (B), and OA (C) (both from reference material), using negative ionization (collision energy: 82); Figure S2. CID fragmentation spectra in the range 100–300 *m*/*z* of Iso-DTX2 (A) (from a mussel sample), DTX2 (B), and OA (C) (both from reference material), using negative ionization (collision energy: 82); Figure S3. CID fragmentation spectra in the range 200–810 *m*/*z* of the H adducts of Iso-DTX2 (A) (from a mussel sample), DTX2 (B), and OA (C) (both from reference material), using positive ionization (collision energy: 40); Figure S4. CID fragmentation spectra in the range 100–300 *m*/*z* of the fragment of 723 *m*/*z* proceeding from the Na adducts of Iso-DTX2 (A) (from a mussel sample), DTX2 (B), and OA (C) (both from reference material), using positive ionization (collision energy: 72); Figure S5. Chromatograms of the transition 803.5 > 255.2 from a DSP-MUS-c certified reference material, from a mussel sample containing Iso-DTX2; Figure S6. Chromatograms of mussel samples of the same toxic episode obtained at the maximum of OA concentration (A) and one month after that (B), showing the increase in importance of Iso-DTX2 and DTX2 relative to OA, due to the lower depuration rate. Table S1. Regression analysis of the relationship between Iso-DTX2 and OA and DTX2 using the data subjected to the transformation log(x + 1).
