# Supplementary material for: A Dinophysistoxin 2-like Okadaic Acid Isomer Present in Bivalves and Water Column: Temporal and Spatial Distribution in the Northwest Iberian Peninsula
Source: Toxins (Basel). 2026 Aug 10;18(8):345. doi: 10.3390/toxins18080345 (PMC13517348; doi:10.3390/toxins18080345)
Supplement: Supplementary file 1 [file toxins-18-00345-s001.zip › toxins-4437503-supplementary.pdf]

Supplementary Materials: A dinophysistoxin 2-like okadaic acid isomer present in bivalves and water column: temporal and spatial distribution in Northwest Iberian Peninsula.

Juan Blanco, Ángeles Moroño, Fabiola Arévalo, Jorge Correa, Araceli E. Rossignoli and Juan Pablo Lamas

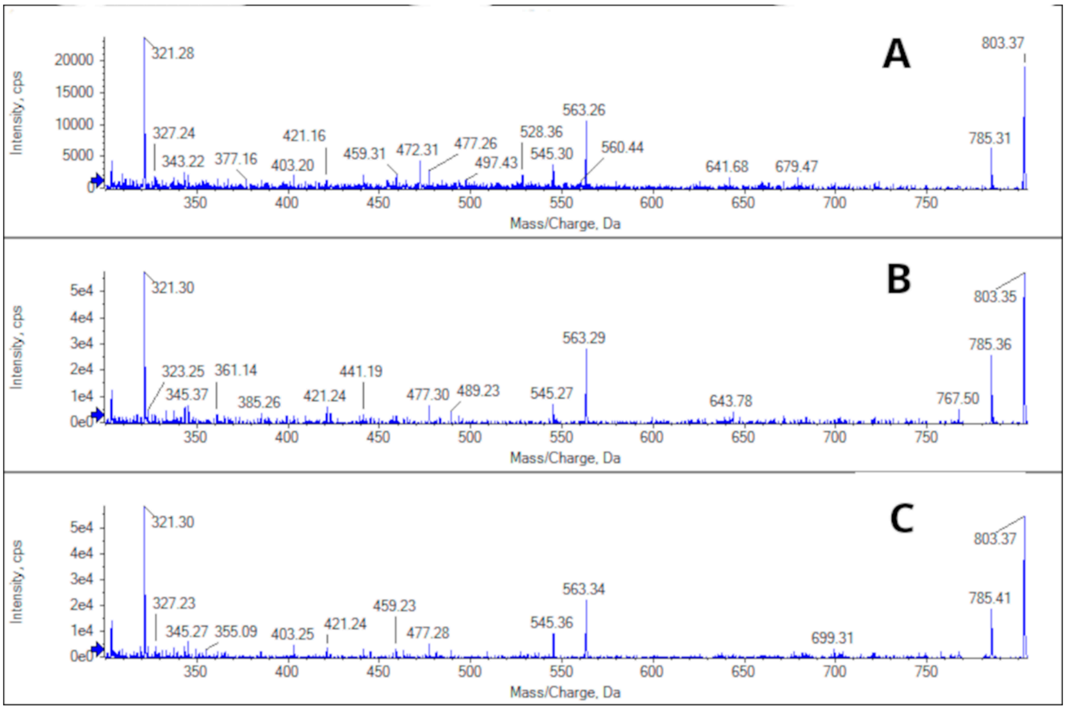

Figure S1. CID fragmentation spectra in the range 300–805 *m*/*z* of Iso-DTX2 (A) (from a mussel sample), DTX2 (B), and OA (C) (both from reference material), using negative ionization (collision energy: 82).

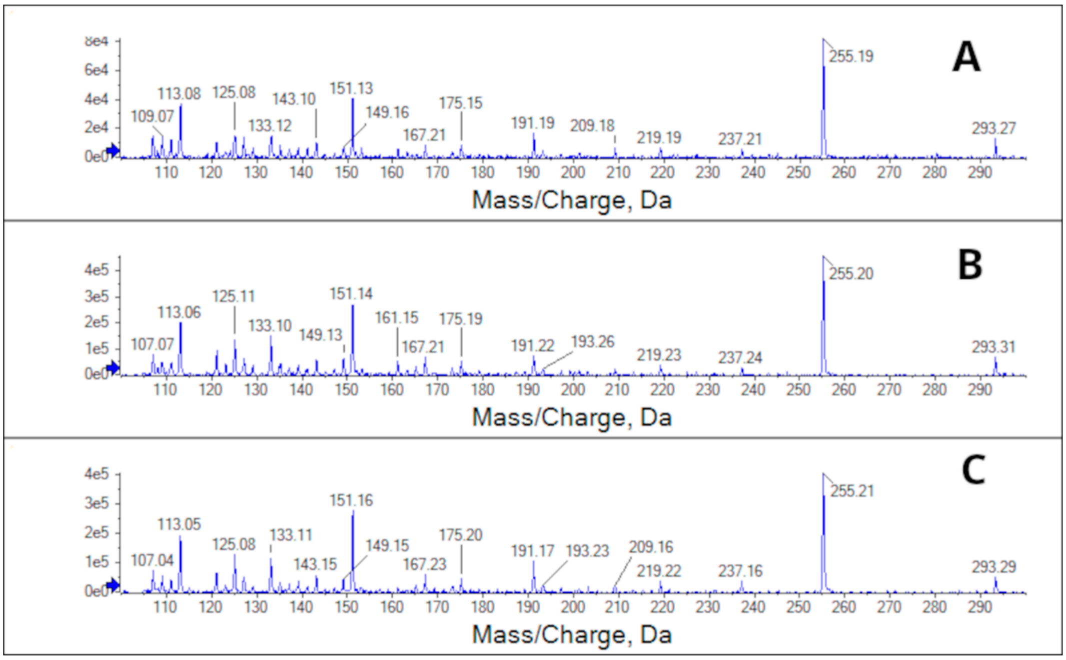

Figure S2. CID fragmentation spectra in the range 100–300 *m/z* of Iso-DTX2 (A) (from a mussel sample), DTX2 (B), and OA (C) (both from reference material), using negative ionization (collision energy: 82).

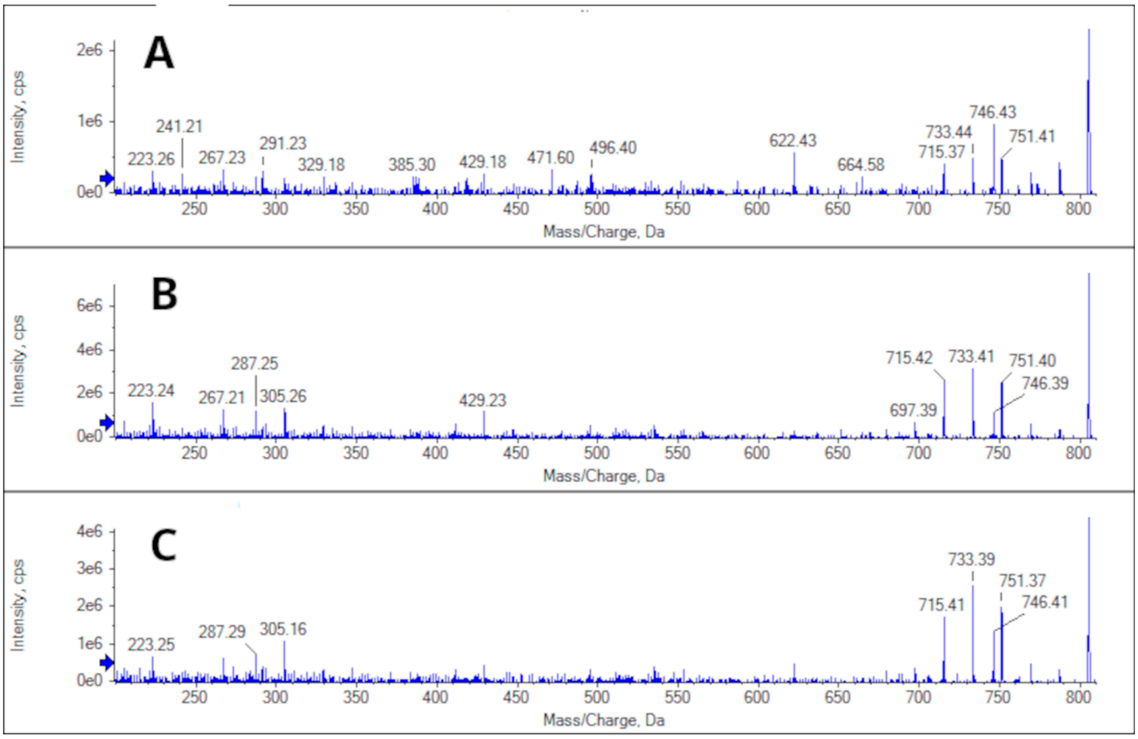

Figure S3. CID fragmentation spectra in the range 200–810 *m/z* of the H adducts of Iso-DTX2 (A) (from a mussel sample), DTX2 (B), and OA (C) (both from reference material), using positive ionization (collision energy: 40).

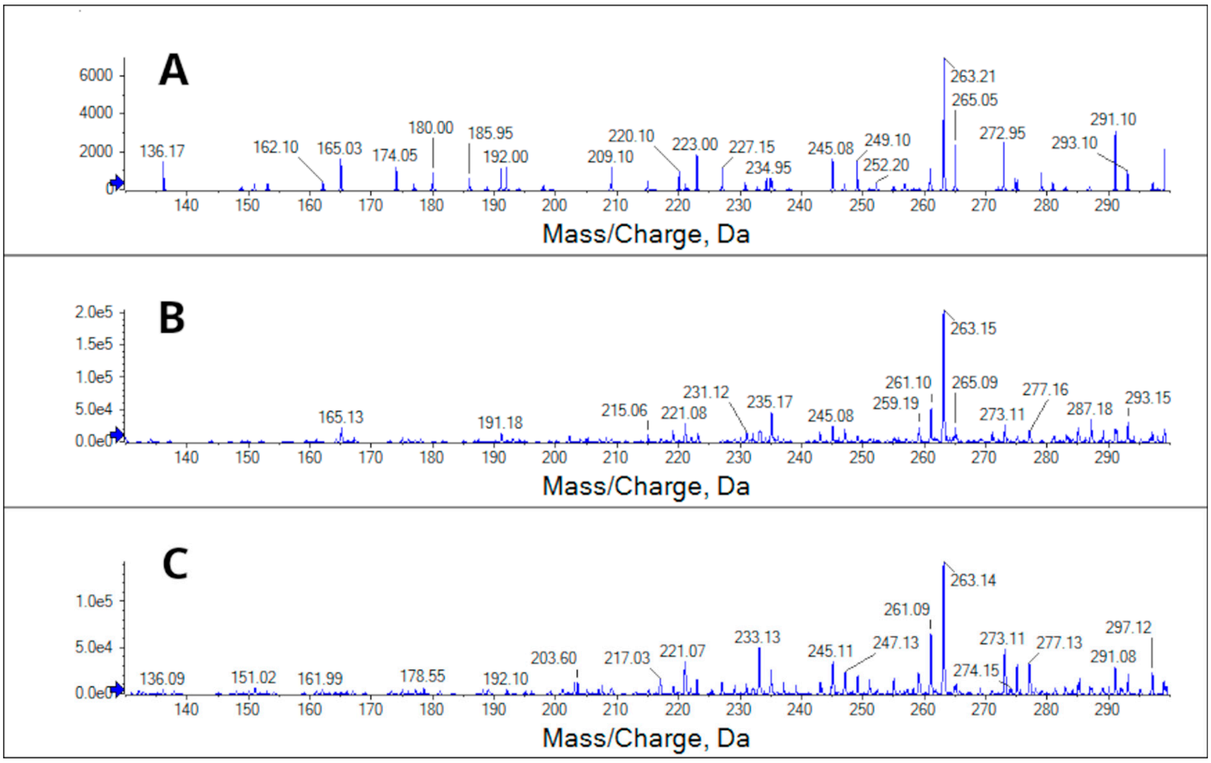

Figure S4. CID fragmentation spectra in the range 100–300 *m*/*z* of the fragment of 723 *m*/*z* proceeding from the Na adducts of Iso-DTX2 (A) (from a mussel sample), DTX2 (B), and OA (C) (both from reference material), using positive ionization (collision energy: 72).

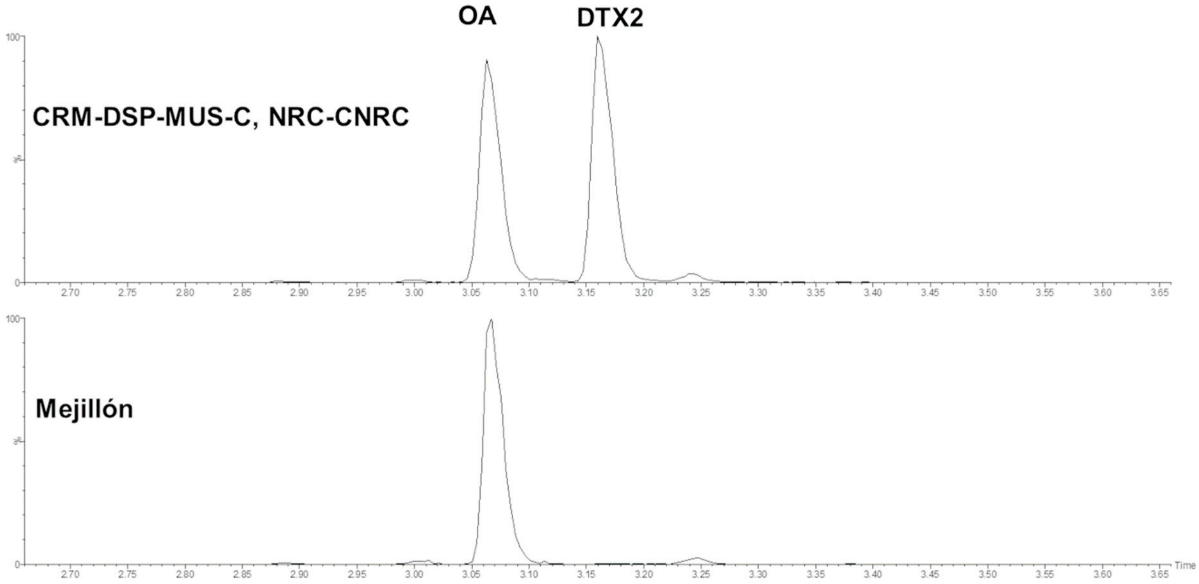

Figure S5. Chromatograms of the transition 803.5 > 255.2 from a DSP-MUS-c certified reference material, from a mussel sample containing Iso-DTX2.

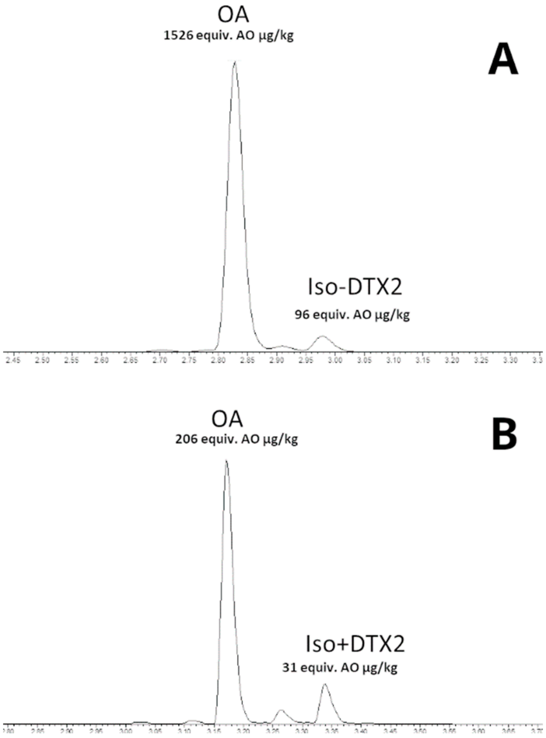

Figure S6. Chromatograms of mussel samples of the same toxic episode obtained at the maximum of OA concentration (A) and one month after that (B), showing the increase in importance of Iso-DTX2 and DTX2 relative to OA, due to the lower depuration rate.

Table S1. Regression analysis of the relationship between Iso-DTX2 and OA and DTX2 using the data subjected to the transformation log(x + 1).

Predictor	Estimate	Std. Error	t value	Pr(>|t|)
(Intercept)	-0.983350	0.036749	-26.76	< 2e-16 ***
logOAm1	0.834398	0.009379	88.97	< 2e-16 ***
logDTX2m1	0.085743	0.016648	5.15	2.64e-07 ***
logOAm1:logDTX2m1	-0.002878	0.004235	-0.68	0.497

Signif. codes: 0 '***' 0.001 '**' 0.01 '*' 0.05 '.' 0.1 ' ' 1

Residual standard error: 0.3536 on 11276 degrees of freedom
Multiple R-squared: 0.6549, Adjusted R-squared: 0.6548
F-statistic: 7133 on 3 and 11276 DF, p-value: < 2.2e-16
